# Supplementary material for: Comparative analysis of syngeneic mouse models of high-grade serous ovarian cancer
Source: Commun Biol. 2023 Nov 13;6:1152. doi: 10.1038/s42003-023-05529-z (PMC10643551; doi:10.1038/s42003-023-05529-z)
Supplement: Supplementary file 2 — Supplementary Information [file 42003_2023_5529_MOESM2_ESM.pdf]

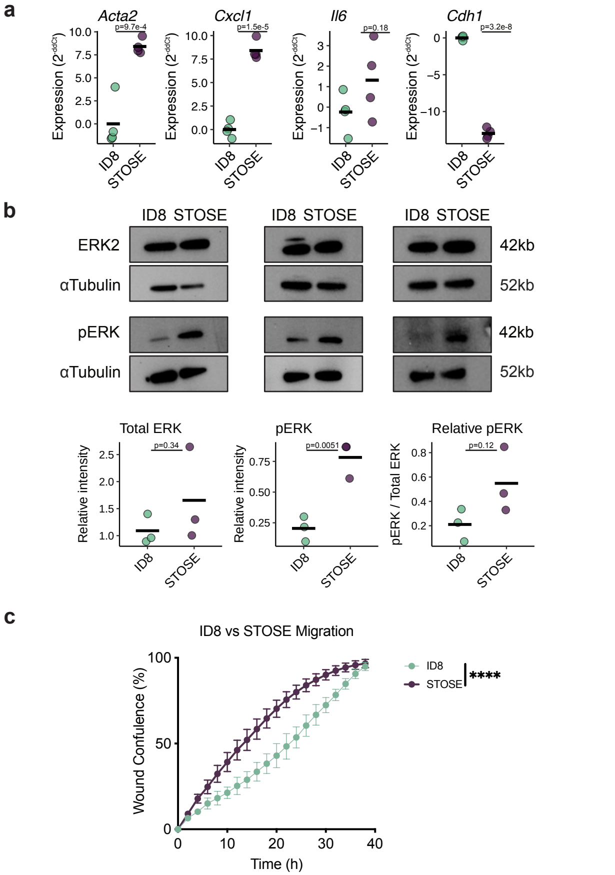

Supplementary Figure 1. Properties of STOSE and ID8 cells. a. Quantitative PCR (qPCR) of select genes validating differential expression measurements from RNA-seq data. 2-ddCt values reflect relative expression, normalized to Ppia and Rplp0. Replicates (n=4) are independent from those used in the RNA-seq experiment. P-values are computed using a Student's t-test. **b.** Top: Western blot analysis of ERK2 and pERK levels in ID8 and STOSE lysates (n=3). Bottom: Densitometric quantification of Total ERK2 and pERK (normalized to αTubulin levels) as well pERK/Total ERK2 ratios. P-values are calculated using a Student's t-test. **c**. Migration rates for STOSE and ID8 cells. Quantifications are based on the percentage of scratch wound closure. n=2 independent experiments, 4 technical replicates per experiment

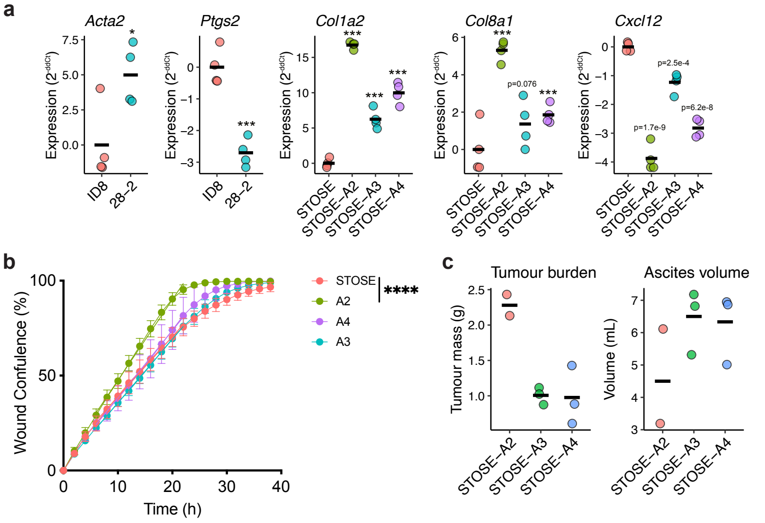

Supplementary Figure 2. Mesenchymal features of ascites-derived cell lines. a. Quantitative PCR (qPCR) of select genes validating differential expression measurements from RNA-seq data. 2-ddCt values reflect relative expression, normalized to Ppia and Rplp0. Replicates (n=4) are independent from those used in the RNA-seq experiment. P-values are computed using a Student's t-test, comparing each sample to the parental cell line. * p<0.05, ** p<0.01, *** p<0.001. **b.** Migration rates for STOSE and ascites derivatives. Quantifications are based on the percentage of scratch wound closure. n=2 independent experiments, 4 technical replicates per experiment. A line of best fit for the linear portion of each curve is included. **** p<0.0001, least-squares regression. **c.** Tumour burden and ascites volume from orthotopic (intrabursal) tumours generated from ascites-derived STOSE cells. n=3, STOSE-A3/4; n=2, STOSE-A2.

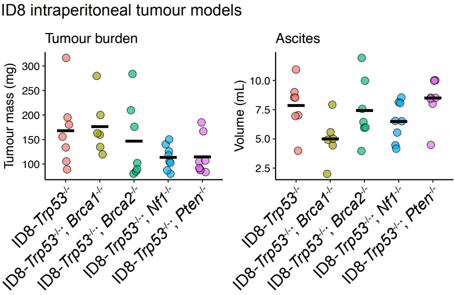

Supplementary Figure 3. Features of intraperitoneal ID8 tumours. Tumour burden and ascites volume from IP tumours generated from ID8 derivate models. Tumour burden measurements are exclusively from tumours of the uterine horn. n=8 per model.

Suppementary Table 1 - qPCR primer sequences

Target	Sequence (5'→ 3')	
	Forward	Reverse
Ppia	AGGGTGGTGACTTTACACGC	GATGCCAGGACCTGTATGCT
36B4	TGACATCGTCTTTAAACCCCG	TGTCTGCTCCCACAATGAAG
Cxcl12	CAGTGACGGTAAACCAGTCAGC	TGGCGATGTGGCTCTCG
Acta2	AGCCATCTTTCATTGGGATGGAG	CATGGTGGTACCCCCTGACA
Cdh1	GGTTTTCTACAGCATCACCG	GCTTCCCCATTTGATGACAC
Il6	CGGAGAGGAGACTTCACAGAG	ATTTCCACGATTTCCCAGAG
Cxcl1	ACCCAAACCGAAGTCATAGCC	TTGTCAGAAGCCAGCGTTCA
Fos	GTGAAGACCGTGTCAGGAGG	GATCTGTCTCCGCTTGGAGT
Fn1	CTGAACCAGCCTACAGATGAC	CATTTTCTCCCTGCCGATCC
Cxcl17	CCCAAAGAGAAAAGCCACAG	GTTTGAGAAATTGCTGGCAGG
Ptgs2	CAAAAGAAGTGCTGGAAAAGGT	GGATGAACTCTCTCCGTAGAAGA
Col1a2	CTTCGTGCCTAGCAACATGC	TGAGCAGCAAAGTTCCCAGT
Col1a8	ACTCTGTCAGACTCATTCAGGC	CAAAGGCATGTGAGGGACTTG
Krt7	CGCCGCTGAGTGTGGACATCG	CTGGCTGCTCTTGGCTGACTTCTG
Star	GCTGTGAAGGCTAAGGGATAAG	GTGACATTTGGAGCTGGTAAGA
