# Supplementary material for: Comparative analysis of syngeneic mouse models of high-grade serous ovarian cancer
Source: Commun Biol. 2023 Nov 13;6:1152. doi: 10.1038/s42003-023-05529-z (PMC10643551; doi:10.1038/s42003-023-05529-z)
Supplement: Supplementary file 3 — Description of Additional Supplementary Files [file 42003_2023_5529_MOESM3_ESM.pdf]

Description of Additional Supplementary Files

File name: Supplementary Data 1
Description: The source data behind the graphs in the figures.

File name: Supplementary Data 2
Description: Gene sets used in the manuscript.
