# Supplementary material for: Comparative analysis of syngeneic mouse models of high-grade serous ovarian cancer
Source: Commun Biol. 2023 Nov 13;6:1152. doi: 10.1038/s42003-023-05529-z (PMC10643551; doi:10.1038/s42003-023-05529-z)
Supplement: Supplementary file 6 — Reporting Summary [file 42003_2023_5529_MOESM6_ESM.pdf]

nature portfolio

Corresponding author(s): David P Cook

Last updated by author(s): Aug 31, 2023



# Reporting Summary

Nature Portfolio wishes to improve the reproducibility of the work that we publish. This form provides structure for consistency and transparency in reporting. For further information on Nature Portfolio policies, see our Editorial Policies and the Editorial Policy Checklist.

## Statistics

For all statistical analyses, confirm that the following items are present in the figure legend, table legend, main text, or Methods section.

| n/a | Confirmed | |
|---|---|---|
| ☐ | ☒ | The exact sample size (*n*) for each experimental group/condition, given as a discrete number and unit of measurement |
| ☐ | ☒ | A statement on whether measurements were taken from distinct samples or whether the same sample was measured repeatedly |
| ☐ | ☒ | The statistical test(s) used AND whether they are one- or two-sided<br>*Only common tests should be described solely by name; describe more complex techniques in the Methods section.* |
| ☒ | ☐ | A description of all covariates tested |
| ☐ | ☒ | A description of any assumptions or corrections, such as tests of normality and adjustment for multiple comparisons |
| ☐ | ☒ | A full description of the statistical parameters including central tendency (e.g. means) or other basic estimates (e.g. regression coefficient) AND variation (e.g. standard deviation) or associated estimates of uncertainty (e.g. confidence intervals) |
| ☐ | ☒ | For null hypothesis testing, the test statistic (e.g. *F*, *t*, *r*) with confidence intervals, effect sizes, degrees of freedom and *P* value noted<br>*Give P values as exact values whenever suitable.* |
| ☒ | ☐ | For Bayesian analysis, information on the choice of priors and Markov chain Monte Carlo settings |
| ☒ | ☐ | For hierarchical and complex designs, identification of the appropriate level for tests and full reporting of outcomes |
| ☐ | ☒ | Estimates of effect sizes (e.g. Cohen's *d*, Pearson's *r*), indicating how they were calculated |

*Our web collection on statistics for biologists contains articles on many of the points above.*

## Software and code

Policy information about availability of computer code

| | |
|---|---|
| Data collection | No software was used to collect data for the study |
| Data analysis | All code required to reproduce analyses and figures are publicly available at https://github.com/dpcook/rna_seq_ovcan |

For manuscripts utilizing custom algorithms or software that are central to the research but not yet described in published literature, software must be made available to editors and reviewers. We strongly encourage code deposition in a community repository (e.g. GitHub). See the Nature Portfolio guidelines for submitting code & software for further information.

## Data

Policy information about availability of data

All manuscripts must include a data availability statement. This statement should provide the following information, where applicable:

- Accession codes, unique identifiers, or web links for publicly available datasets
- A description of any restrictions on data availability
- For clinical datasets or third party data, please ensure that the statement adheres to our policy

All RNA-seq data is available on NCBI GEO at the accession GSE242835

## Human research participants

Policy information about studies involving human research participants and Sex and Gender in Research.

| | |
|---|---|
| Reporting on sex and gender | *Use the terms sex (biological attribute) and gender (shaped by social and cultural circumstances) carefully in order to avoid confusing both terms. Indicate if findings apply to only one sex or gender; describe whether sex and gender were considered in study design whether sex and/or gender was determined based on self-reporting or assigned and methods used. Provide in the source data disaggregated sex and gender data where this information has been collected, and consent has been obtained for sharing of individual-level data; provide overall numbers in this Reporting Summary. Please state if this information has not been collected. Report sex- and gender-based analyses where performed, justify reasons for lack of sex- and gender-based analysis.* |
| Population characteristics | *Describe the covariate-relevant population characteristics of the human research participants (e.g. age, genotypic information, past and current diagnosis and treatment categories). If you filled out the behavioural & social sciences study design questions and have nothing to add here, write "See above."* |
| Recruitment | *Describe how participants were recruited. Outline any potential self-selection bias or other biases that may be present and how these are likely to impact results.* |
| Ethics oversight | *Identify the organization(s) that approved the study protocol.* |

Note that full information on the approval of the study protocol must also be provided in the manuscript.

# Field-specific reporting

Please select the one below that is the best fit for your research. If you are not sure, read the appropriate sections before making your selection.

☒ Life sciences ☐ Behavioural & social sciences ☐ Ecological, evolutionary & environmental sciences

For a reference copy of the document with all sections, see nature.com/documents/nr-reporting-summary-flat.pdf

# Life sciences study design

All studies must disclose on these points even when the disclosure is negative.

| | |
|---|---|
| Sample size | No sample size calculation was performed. A minimum of 3 biological replicates were used for all experiments as per field standards. |
| Data exclusions | No data was excluded |
| Replication | A minimum of 3 independent biological replicates were used for all experiments. A validation experiments were performed on samples independent from those used for RNA-seq analysis |
| Randomization | Mice were randomized for all animal experiments. Batching procedures were avoided to prevent batch effects. |
| Blinding | Blinding was not performed for the study. |

# Reporting for specific materials, systems and methods

We require information from authors about some types of materials, experimental systems and methods used in many studies. Here, indicate whether each material, system or method listed is relevant to your study. If you are not sure if a list item applies to your research, read the appropriate section before selecting a response.

### Materials & experimental systems

| n/a | Involved in the study | |
|---|---|---|
| ☐ | ☒ | Antibodies |
| ☐ | ☒ | Eukaryotic cell lines |
| ☒ | ☐ | Palaeontology and archaeology |
| ☐ | ☒ | Animals and other organisms |
| ☒ | ☐ | Clinical data |
| ☒ | ☐ | Dual use research of concern |

### Methods

| n/a | Involved in the study | |
|---|---|---|
| ☒ | ☐ | ChIP-seq |
| ☒ | ☐ | Flow cytometry |
| ☒ | ☐ | MRI-based neuroimaging |

## Antibodies

| | |
|---|---|
| Antibodies used | anti-ERK2 (1:2000, SC-154, Santa cruz)<br>anti-pERK (1:2000, SC-7383, Santa Cruz)<br>anti-mouse (1:10000, ab6728, Abcam)<br>anti-rabbit (1:10000, 711-035-152, Jackson Immunoresearch)<br>anti-aTubulin (1:1000, 2144S, Cell Signalling) |
| Validation | Antibodies were tested on a variety of cancer cell lines. Molecular weight of protein bands were compared to expected size. |

## Eukaryotic cell lines

Policy information about cell lines and Sex and Gender in Research

| | |
|---|---|
| Cell line source(s) | ID8 cells were provided by Dr. Kathy Roby<br>ID8-Trp53 -/--C3, ID8-Trp53-/;-Brca1-/-, 376 ID8-Trp53-/-;Brca2-/-, ID8-Trp53-/-;Nf1-/-, ID8-Trp53-/-;Pten-/- were provided by Dr. Iain McNeish<br>MOE-Pten-shRNA, MOE-Pten-shRNA;TP53R273H, and MOE-Pten-shRNA;KRASG12V were provided by Dr. Joanna Burdette<br>All other lines were developed by the authors |
| Authentication | To ensure the validity of cell lines, we employed several practices. First, the capacity to form tumours in immune-competent mice ensures that the cells are of the appropriate strain. Engineered modifications are assessed by gennotyping or Western blot. Finally, only low passage cells (split fewer than approximately 10 times following their receipt) are used for experiments, reducing the effects of phenotypic or genetic drift. |
| Mycoplasma contamination | All lines were tested and confirmed negative for mycoplasma contamination |
| Commonly misidentified lines<br>(See ICLAC register) | *Name any commonly misidentified cell lines used in the study and provide a rationale for their use.* |

## Animals and other research organisms

Policy information about studies involving animals; ARRIVE guidelines recommended for reporting animal research, and Sex and Gender in Research

| | |
|---|---|
| Laboratory animals | FVB/N or C57BL/6 female mice aged 8-10 weeks were ordered from Charles River Laboratories and were used for all experiments. |
| Wild animals | *Provide details on animals observed in or captured in the field; report species and age where possible. Describe how animals were caught and transported and what happened to captive animals after the study (if killed, explain why and describe method; if released, say where and when) OR state that the study did not involve wild animals.* |
| Reporting on sex | Only female mice were used for the study as they are models of ovarian cancer |
| Field-collected samples | *For laboratory work with field-collected samples, describe all relevant parameters such as housing, maintenance, temperature, photoperiod and end-of-experiment protocol OR state that the study did not involve samples collected from the field.* |
| Ethics oversight | Animal experiments to generate orthotopic tumours were carried out using protocols approved by the Animal Care Committee at the University of Ottawa and conforming to the standards defined by the Canadian Council on Animal Care (CCAC). Procedures for intraperitoneal tumour models were approved by the University Animal Care Committee (UACC) at Queen's University and the CCAC. |

Note that full information on the approval of the study protocol must also be provided in the manuscript.
